# A quantile-based method for association mapping of quantitative phenotypes: an application to rheumatoid arthritis phenotypes

**DOI:** 10.1186/1753-6561-3-s7-s18

**Published:** 2009-12-15

**Authors:** Saurabh Ghosh, Krishna Rao Sanapala, Abhik Ghosh, Sujatro Chakladar

**Affiliations:** 1 Human Genetics Unit, Indian Statistical Institute, Kolkata, India

## Abstract

Genetic association of population-based quantitative trait data has traditionally been analyzed using analysis of variance (ANOVA). However, violations of certain statistical assumptions may lead to false-positive association results. In this study, we have explored model-free alternatives to ANOVA using correlations between allele frequencies in the different quantile intervals of the quantitative trait and the quantile values. We performed genome-wide association scans on anti-cyclic citrullinated peptide and rheumatoid factor-immunoglobulin M, two quantitative traits correlated with rheumatoid arthritis, using the data provided in Genetic Analysis Workshop 16. Both the quantitative traits exhibited significant evidence of association on Chromosome 6, although not in the human leukocyte antigen region which is known to harbor a major gene predisposing to rheumatoid arthritis. We found that while a majority of the significant findings using the asymptotic thresholds of ANOVA was not validated using permutations, a relatively higher proportion of the significant findings using the asymptotic cut-offs of the correlation statistic were validated using permutations.

## Background

Complex genetic traits are usually characterized by correlated quantitative precursors. Because quantitative traits carry more information on within-genotype variation compared to binary (affected/unaffected) traits, it has been argued that analyzing quantitative endophenotypes may be a more powerful strategy than analyzing clinical end-points for identifying genes controlling a complex trait. While some family-based methods of association have been developed for quantitative traits [1-3], population-based quantitative trait data have usually been analyzed using classical analysis of variance (ANOVA) methods. However, ANOVA is valid in a strict statistical sense only under the assumption of normality of the variable of interest and equality of variances in each underlying group. On the other hand, the assumption of normality and equality of variances of the quantitative traits for the different genotypes at a quantitative trait locus (QTL) is genetically unrealistic, particularly if the trait is correlated with a disease outcome [4]. Studies have shown that the ANOVA statistic may lead to an inflated rate of false positives when the underlying assumptions are violated [5]. Thus, it is of interest to explore for model-free alternatives that would circumvent this problem. In this study, we explore some novel quantile-based statistics to test for allelic association and perform genome-wide association scans of anti-cyclic citrullinated peptide (anti-CCP) and rheumatoid factor immunoglobulin M (RFUW) levels, two quantitative phenotypes correlated with the rheumatoid arthritis (RA) affection status in the North American Rheumatoid Arthritis Consortium (NARAC) data provided in Genetic Analysis Workshop (GAW) 16. The basic paradigm of the methods is that a marker allele in linkage disequilibrium with an allele at the QTL would have either a strictly increasing or a strictly decreasing frequency distribution across the range of quantitative trait values. We compare the association results of our proposed methods with those obtained using ANOVA.

## Data description

For our analyses, we used data on anti-CCP and RFUW levels along with genotypes at 531,689 single-nucleotide polymorphisms (SNPs) distributed over the 22 autosomal chromosomes. Data on the two quantitative traits were available only on individuals affected with RA. After removing the individuals with missing phenotype data, our methods used data on 867 individuals (640 females and 227 males) for anti-CCP and 746 individuals (548 females and 148 males) for RFUW. We found that 51,524 SNPs had a minor allele frequency less than 0.05. Because these markers are unlikely to be involved in disease pathogenesis under a common-variant, common-disease (CVCD) model, they were also removed from the association analyses.

## Statistical methodology

Suppose *Q*_1_, *Q*_2_,..., *Q*_*k *_denote the *k *sample quantiles of the quantitative trait in study with *Q*_0 _defined as the minimum value of the trait in the data. Suppose *p*_*i *_denotes the proportion of the minor allele at a particular SNP among individuals with quantitative trait values in the interval (*Q*_*i*-1_, *Q*_*i*_), *i *= 1,2,..., k. It can be shown that the frequency of a marker allele conditioned on the value of the quantitative trait lying in an interval (*a*, *b*) is independent of *a *and *b *if and only if the coefficient of linkage disequilibrium between the QTL and the marker locus is zero. Moreover, in the presence of linkage disequilibrium between the two loci, the frequency of the marker allele in positive linkage disequilibrium with the QTL allele predisposing to high values of the quantitative trait will increase with the quantile intervals. Thus, one can test for allelic association based on the correlation between *Q*_*i *_and *p*_*i *_values. A test based on the direct correlation coefficient (*r*) between the two variables would be equivalent to testing whether the slope coefficient of a linear regression of *p*_*i *_values on *Q*_*i *_values is zero or not (that is, whether there is a linear trend (either increasing or decreasing) of *p*_*i *_values with increasing values of *Q*_*i*_). The test statistic is √(*n*-2)|*r*|/√(1-*r*^2^) and is distributed as *t *with (*n-*2) degrees of freedom under the assumption of bivariate normality of the underlying variables, although the assumption can be relaxed for large samples. We note that this statistic is identical to that used for testing the slope coefficient in the linear regression mentioned above. A more robust statistic is based on the rank correlation (*R*) between the variables given by √(*n*-1)|*R*| and is asymptotically distributed as a standard normal variate [6]. However, it may be more appropriate not to rely on the asymptotic distributions and use permutation principles, instead, to determine the significance of the above tests. We randomly permute the quantitative trait values across the individuals keeping the SNP genotype data unchanged. This preserves the marginal distributions of both the quantitative traits and the genotypes, but generates the null distribution of no association. Since it is becoming increasingly evident that the Bonferroni correction for multiple testing is highly over-conservative and hence leads to an elevated rate of false negatives, we use the false discovery rate (FDR) procedure [7] with an overall rate of 0.05 to identify SNPs significantly associated with the quantitative trait in study.

One of the statistical issues is the choice of an appropriate number of quantiles. The method involves the estimation of the minor allele frequency in each quantile interval as well as computing the correlation between these estimated frequencies and the quantile values. If the number of quantiles is too large, each quantile interval will comprise very few observations, leading to inefficient estimation of the allele frequencies. On the other hand, if the number of quantiles is too small, the total number of observations for the computation of the correlation coefficient will not be sufficiently large to make reliable inferences on association. We suggest that each quantile interval comprise approximately 25 observations so that the variance of the estimated allele frequency [*p*(*1*-*p*)/*n*] cannot exceed 0.01. The minimum number of quantiles should be about 30 for appropriate use of large sample properties of the correlation coefficient.

## Results

We first tested for possible deviation from Hardy-Weinberg equilibrium (HWE) for each of the 480,165 SNPs. To attain an overall significance level of 0.05, we used a Bonferroni corrected *p*-value less than 10^-7 ^as evidence of departure from HWE. Those SNPs that failed the HWE test were removed from the association analyses. We performed three tests of association: the first using ANOVA, the second using the direct correlation coefficient, and the third using the rank correlation coefficient for each of the two quantitative traits: anti-CCP and RFUW levels. To determine an effective number of independent tests for an appropriate FDR correction, we used the software HAPLOVIEW, which identified 367,392 tag SNPs, based on a threshold of pairwise *r*^2 ^greater than 0.8.

Neither of the two quantitative traits follows a normal distribution (*p*-value < 0.01 using the Kolmogorov-Smirnov statistic and validated by Q-Q plots). There was also no sex effect in either anti-CCP levels (*p *= 0.109 using a two sample *t*-test, *p *= 0.118 using the Mann-Whitney test) or RFUW levels (*p *= 0.7 using a two sample *t*-test, *p *= 0.08 using the Mann-Whitney test) and hence, no sex adjustment was necessary for the association analyses. For the quantile-based methods, we divided the data into 31 quantile intervals for both the quantitative traits (for anti-CCP, 28 observations in each of the first 30 intervals and 27 in the last interval; for RFUW, 24 observations in each of the first 30 intervals and 26 in the last interval).

We performed our association analyses on all the 22 chromosomes using ANOVA and the two quantile-based statistics. The most significant association findings are presented in Table 1. Analyses on anti-CCP levels using ANOVA provided 935 significantly associated SNPs. The corresponding numbers from the direct correlation coefficient and rank correlation statistics were 180 and 0, respectively. The most extreme *p*-value from the rank correlation statistic was 0.0036 on chromosome 11. There were two genomic regions where multiple SNPs exhibited significant evidence of association: one in the 6q22 region (124,503,495-124,568,348 bp), which contains the *NKAIN2 *(sodium/potassium transporting ATPase interacting 2) gene and the other in the 11q12 region (38,983,737-39,220,545 bp) containing a psuedogene LOC100129670. We note that another contribution from our GAW group also found significant evidence of association in the 6p22 region with anti-CCP [8].

**Table 1:** Most significant association findings

Phenotype	Method	Chromosome	SNP	*p*-value
Anti-CCP	ANOVA	1	rs1211759	6.58 × 10^-23^
	Correlation	1	rs17123469	5.31 × 10^-19^
RFUW	ANOVA	6	rs6456834	7.07 × 10^-23^
	Correlation	1	rs2785665	1.03 × 10^-19^

When we analyzed RFUW levels using ANOVA, we found 1399 significantly associated SNPs, while the direct correlation coefficient and the rank correlation statistics provided 178 and 15 significant SNPs, respectively. The most extreme *p*-value based on the rank correlation statistic was 0.006 on chromosome 2. However, unlike anti-CCP, the RFUW levels did not exhibit any genomic region with a cluster of significant SNPs.

As pointed out in the section entitled "Statistical Methodology," it was of interest to explore whether the above association findings, obtained using asymptotic tests, can be validated by permutation tests. We found that for anti-CCP, only 182 of the 935 SNPs that showed significant evidence of association using ANOVA were validated by permutations, while for RFUW, only 297 of the 1399 SNPs showed significant association using permutations. However, the corresponding numbers using the direct correlation statistic were 47 out of 180 SNPs for anti-CCP and 99 out of 178 for RFUW. Thus, among the SNPs exhibiting significant association using asymptotic thresholds, the permutation tests validated a higher proportion of SNPs for the correlation statistic compared to ANOVA. This can be intuitively explained by the fact that the quantile-based correlation statistic is model-free in nature and hence relatively more robust to violations in distributional assumptions. The rank correlation statistic did not provide any significant evidence of association using the permutation test procedure.

None of the SNPs in the two regions that have been implicated in RA pathogenesis, the human leukocyte antigen (HLA) region (on 6p21) and the *PTPN22 *gene (on 1p13), showed any significant evidence of association with either of the two quantitative phenotypes at the overall FDR of 0.05 using any of the methods.

## Conclusion

The proposed quantile-based statistics are model-free alternatives to the ANOVA approach for association mapping of quantitative traits and circumvent the problem of departures from normality and homoskedasticity of the quantitative traits conditioned on the QTL genotypes. While these methods are more robust than ANOVA, they are expected to be less powerful when the underlying assumptions of ANOVA are valid. In particular, the rank correlation statistic is bounded by √(*n*-*1*), where *n *is the number of observations, and hence, has limits to the *p*-values when the asymptotic distribution is used. The direct correlation coefficient statistic, which is equivalent to the test of the slope coefficient in a linear regression, is a more suitable alternative. However, a permutation strategy is likely to provide more accurate *p*-values compared to asymptotic distributions, especially if the number of quantiles is not very large. We also note that it is not possible to assess the relative performances of the different methods using real data efficiently. We are carrying out extensive simulations independently to compare the powers of the different tests. Our preliminary simulations based on a sample size of 500 suggest that the powers of the correlation-based statistic and ANOVA are comparable under a homoskedastic QTL model, but the correlation-based method is more powerful under a heteroskedastic model.

An intriguing result of the present study has been the lack of evidence of significant association in the HLA region on chromosome 6 and the *PTPN22 *gene on chromosome 1 with either of the two quantitative phenotypes. A similar phenomenon was also observed in the analyses of these two quantitative traits in the data provided in Genetic Analysis Workshop 15 [9]. This raises the possibility that the biological pathway involved in modulating the levels of anti-CCP and RFUW may be different from that of the clinical end-point of RA. Alternatively, the paradox can be explained by the fact that since a clinical end-point is usually a function of multiple quantitative precursors, the effect size of any gene is small for individual quantitative traits and hence require much larger sample sizes to exhibit significant evidence of association.

Because the data on the two quantitative traits were available only on individuals affected with RA, the association findings should be interpreted as polymorphisms involved in differential elevations of anti-CCP and RFUW levels in RA-affected individuals from the mean normal levels of these phenotypes. There is reduced variation in the quantitative phenotypes when restricted only to RA cases and hence, an appropriate design to identify polymorphisms associated with the two quantitative phenotypes would include data on controls in addition to the cases.

## List of abbreviations used

ANOVA: Analysis of variance; anti-CCP: Anti-cyclic citrullinated peptide; CVCD: Common-variant, common-disease; FDR: False discovery rate; HLA: Human leukocyte antigen; HWE: Hardy-Weinberg equilibrium; GAW16: Genetic Analysis Workshop 16; NARAC: North American Rheumatoid Arthritis Consortium; QTL: Quantitative trait locus; RA: rheumatoid arthritis; RFUW: Rheumatoid factor immunoglobulin M; SNP: Single-nucleotide polymorphism.

## Competing interests

The authors declare that they have no competing interests.

## Authors' contributions

SG conceived the statistical method and drafted the manuscript. KRS managed the data and wrote the computer codes for the statistical methods. AG and SC developed the permutation testing procedure and carried out the statistical analyses. All authors read and approved the final manuscript.
